# Characterization and Classification of Berry (Aronia, Haskap, and Goji) Fruits with High Bioactive Value Grown in Spain

**DOI:** 10.3390/foods13244122

**Published:** 2024-12-20

**Authors:** María Concepción Ayuso-Yuste, Francisco Javier Cruz Calero, María Ramos García, Noelia Nicolás Barroso, María Belén Ramos Alguijo, María José Rodríguez Gómez, Patricia Calvo Magro

**Affiliations:** 1 Agricultural Engineering School, Universidad de Extremadura, Avda. Adolfo Suárez s/n, 06007 Badajoz, Spain; cayuso@unex.es (M.C.A.-Y.); fcruzcal@alumnos.unex.es (F.J.C.C.); 2 Centro de Agricultura Ecológica y de Montaña, Centro de Investigaciones Científicas y Tecnológicas de Extremadura (CICYTEX), Avda. España, nº 43, Plasencia, 10600 Cáceres, Spain; maria.ramos@juntaex.es (M.R.G.); noe.n.b16@gmail.com (N.N.B.); 3 Instituto Tecnológico Agroalimentario de Extremadura, Centro de Investigaciones Científicas y Tecnológicas de Extremadura (CICYTEX), Avda. Adolfo Suárez, s/n, 06007 Badajoz, Spain; mariabelen.ramosa@juntaex.es (M.B.R.A.); patricia.calvo@juntaex.es (P.C.M.)

**Keywords:** antioxidant activity, carotenoids, phenolic compounds, physicochemical parameters, sugar and organic acids, PCA

## Abstract

Aronia, haskap, and goji berries are characterized by their high content of bioactive compounds and their beneficial health properties as well as their resistance to harsh agronomic conditions. In this work, cultivars of these species growing in a mountainous region of central-western Spain were characterized by analyzing physicochemical parameters and bioactive compounds. Goji fruits showed the highest total soluble solid content and the lowest acidity values. The sugar profile suggested that goji cultivars will have a higher sweetness due to higher fructose and glucose content. However, aronia cultivars will be the least sweet due to their high sorbitol content. The total organic acid content was much higher in aronia and haskap than in goji fruits, and the profile varied according to species. The total phenolic content was significantly higher in aronia fruits. A total of 15 phenolic compounds were detected, with anthocyanins being predominant in aronia and haskap berries; however, they were not detected in goji fruits. Nevertheless, carotenoid compounds were found in goji berries and not detected in aronia and haskap fruits. Aronia fruits showed the highest antioxidant capacity compared to haskap and goji fruits. The PCA analysis classified the samples to determine which parameters have the greatest influence.

## 1. Introduction

Red fruits are currently in high demand due to their known health-promoting properties. Thus, they are a good source of many bioactive ingredients, such as organic acids, phenolics, and sugars (glucose, fructose) [1] and nutrients, including vitamins, minerals, dietary fiber, and antioxidants [2]. Therefore, the acceptability and consumption of them has experienced significant increases [3]. Currently, the most consumed berries belong to species of several families such as Rosaceae (strawberry, raspberry, blackberry, and sweet cherry) and Ericaceae (blueberry, cranberry). However, other minority red fruit crops such as aronia, haskap, and goji are emerging as promising alternatives due to their health properties and the need to diversify crops, which is essential for resilience to climate change [4]. These are crops that withstand harsh growing conditions well and could represent an opportunity for diversification in mountain farming areas [5]. However, the qualitative and quantitative composition will vary with the species, cultivar, and agro-environmental conditions [6].

Aronia (*A. melanocarpa* (Michx.) Elliott), also known as black chokeberry, is a hardy shrub native to North America. This fruit has been used against diseases by Native Americans for centuries, and after World War II, aronia cultivation began to become widespread in Europe and Russia. Aronia fruits show a high antioxidant capacity due to their richness in bioactive compounds, especially phenolic compounds such as proanthocyanidins, anthocyanidins, and other flavonoids and phenolic acids [7,8].

On the other hand, the cultivation of haskap (*Lonicera carerulea* L.) is quite recent in the red fruit sector. It is a rustic shrub, which grows spontaneously in humid or mountainous regions of northern Asia and has been used in natural medicine in its places of origin [9]. Although its cultivation is gaining importance in recent years, it is not yet widespread in the European territory, as it has been considered a novel food in the European Union since 2018 [10]. Their healthy properties are due, as in other berries, to their high concentration of physiologically active polyphenols, including flavonoids and phenolic acids [11,12].

Goji berries (*Lycium barbarum* L.) are the fruits of a shrub that grows spontaneously in northwestern China and other areas of Asia and is now cultivated in all parts of the world, including the Mediterranean area [13,14]. Its use in Chinese medicine is very ancient, and it is recognized for its diverse biological and antioxidant activities related to health promoting properties [14]. As for the other berries mentioned above, the health properties of Goji berries are due to their content of polyphenol compounds, but they also possess carotenoids, which are yellow-red antioxidant pigments, responsible for its color, and their consumption has also been linked to eye health benefits [13].

As for the consumption of these fruits, only goji berries can be consumed fresh, although they are usually taken dehydrated or transformed into juices, liqueurs or ingredient infusions [15]. Aronia and haskap berries are very acidic, astringent, somewhat bitter and unpleasant in the mouth, so they are processed into juices or extracts for the pharmaceutical industry or nutritional supplementation [16,17].

In order to know the overall quality and functional potential of different cultivars of aronia, haskap, and goji berries grown in the central-west region of Spain, bioactive compounds, such as organic acids, sugars, and phenolic and carotenoid compounds; antioxidant activity; and physicochemical parameters, such as soluble solid content, pH, and titratable acidity, were analyzed in this work. In addition, a principal component analysis was applied to classify the samples and to determine which parameters influence this classification.

## 2. Materials and Methods

### 2.1. Plant Material

Aronia berry (*Aronia melanocarpa* (Michx.) Elliott.) fruits of ‘Nero’, ‘Viking’, and ‘Galicjanka’ cultivars; haskap berry (*Lonicera caerulea* L.) fruits of ‘Blue Velvet’ cultivar; and goji berry (*Lycium barbarum* L.) fruits of ‘Turgidus’ and ‘New Big’ cultivars were collected at commercial maturity in 2023 from 3-year-old berries plant from the CICYTEX experimental field, located in Malpartida de Plasencia (Extremadura, Spain) (39°54′31.7″ N, 5°55′18.4″ W), with an inland Mediterranean climate. The fruits of each cultivar were harvested at a state of commercial maturity defined, in each case, by external color and uniformity of fruit size. The plant material belongs to varietal collections composed of entries of a homogeneous species from different geographical areas. The plants were grown under organic management, with a drip irrigation system and fertilization through a winter application of compost and several foliar applications with algae biostimulants during the vegetative period. Pest control was carried out with organic products (sulphur, organic oils, and natural pyrethrins). The collected fruits were immediately refrigerated (2 ± 1 °C) and transported to the CICYTEX-INTAEX facilities. The physicochemical parameters of all samples were analyzed on the same day. Subsequently, the samples were frozen at −80 °C in hermetic containers for better preservation of the bioactive compounds and to avoid moisture loss.

### 2.2. Physicochemical Analysis

The moisture content was measured gravimetrically by drying approximately 1.0 g of samples at 105 °C for 24 h. A portable digital refractometer Pal-1 (ATAGO Co., Ltd., Tokyo, Japan) was used to measure the total soluble solids (TSS) from a homogenate of thirty fruits. The pH and titratable acidity (TA) were determined using an automatic titrator T-50 Graphix (Mettler Toledo, Barcelona, Spain). For it, three grams of homogenate were mixed with 60 mL of deionized water and titrated with sodium hydroxide 0.1 N to a final pH of 8.1. The results are expressed as g malic acid 100 g^−1^ of fresh sample. Maturation index (MI) was calculated as TSS/TA.

### 2.3. Sugars

The sugar profile was determined following the method described by Magro et al. [18] by extraction of 1 g of sample with 10 mL of Milli-Q water and subsequent centrifugation at 12,000 rpm for 10 min. The supernatant was filtered through a 0.45 µm Filter-Lab (Agilent Technologies, Madrid, Spain) to be analyzed. An Agilent 1200 liquid chromatograph coupled with a refractive index detector (RID) (G-1362) (Agilent Technologies, Santa Clara, CA, USA) was used for the analysis. Separation of the sugars was achieved in a RezexTM RPM-Monosaccharide PB + 2 (300 × 7.8 mm, 8 μm) column at 75 °C. The mobile phase was water with a flow rate of 0.6 mL min^−1^ according to the method described by Phenomenex^®^ (https://www.phenomenex.com/Application/Detail/5508?returnURL=/Application/Search&fsr=1, accessed on 1 July 2024) for saccharide determination. Sugar standards (glucose, fructose, and sucrose) were obtained from Sigma Aldrich (St. Louis, MO, USA). The results are expressed as g sugar 100 g^−1^ of fresh weight.

### 2.4. Organic Acids

The organic acid profile was carried out according to Magro et al. [18] by extraction of 1 g of homogenized samples with 10 mL of Milli-Q water and sonication in an ultrasonic bath (P-Selecta Model 516, Barcelona, Spain) at 35 kHz frequency for 30 min at room temperature. The extracts were clarified by centrifugation at 10,000 rpm for 10 min. The supernatant was filtered through a 0.45-μm nylon Filter-Lab (Agilent Technologies, Spain) to be analysed. Subsequently, a liquid chromatograph (Agilent 1200, Agilent Technologies, CA, USA) coupled with a diode array detection (DAD) (measuring at 210 nm) were employed according to Phenomenex^®^ methods (https://www.phenomenex.com/applications/single?appid=18812, accessed on 1 July 2024). A Rezex ROA-Organic Acid column (300 × 7.8 mm, 8 μm) at 55 °C, with a mobile phase of 0.005 N sulphuric acid and a flow rate of 0.5 mL min^−1^ was employed. The organic acid standards were obtained from Sigma-Aldrich (St. Louis, MO, USA). Results are expressed as g organic acid 100 g^−1^ of fresh weight.

### 2.5. Total Phenolic Content and Antioxidant Activity

The total phenolic content (TPC) was determined using the method described by Fatchurrahman et al. [19]. For this, 1 g of sample was homogenized with 30 mL of a water/methanol solution (20:80) and 2 mmol L^−1^ sodium fluoride in Ultra Turrax for 1 min. Then, the mixture was centrifuged at 9056× *g* for 10 min at 4 °C. For the reaction development, 100 µL of the extract was taken and mixed with 1.58 mL of water, 100 µL of Folin–Ciocalteu reagent (Panreac Applichem, Barcelona, Spain), and 300 µL of sodium carbonate solution (200 g L^−1^). The absorbance was read at 725 nm after 2 h using a Shimadzu UV-Vis spectrophotometer (Kyoto, Japan). A calibration curve using gallic acid (Sigma Aldrich, St. Louis, MO, USA) was used for the quantification of total phenolic compounds. Results are expressed as mg gallic acid equivalents per g fresh weight (mg GAE g^−1^ fw).

The antioxidant activity assay was performed according to Capotorto et al. [20] with minor modifications. For it, 25 µL of the previous extract was mixed with 0.950 mL of DPPH solution, and after incubation for 1 h, the absorbance was read at 515 nm. Trolox (Sigma Aldrich, St. Louis, MO, USA) was used as a standard, and the antioxidant activity is expressed as mg Trolox equivalents (TE) g^−1^ fw.

### 2.6. Individual Phenolic Compounds

The individual phenolic compounds were determined following the method described by Manzano et al. [21]. For it, 10 µL of the extract obtained for the analysis of total phenolic compounds were injected into an 1100 Series HPLC system (Agilent Technologies, Santa Clara, CA, USA) equipped with a DAD and fluorescence detector. The identification of the compounds was performed by comparing the retention times and purity peak spectra with stock dissolution. Quantification was performed by chromatographic comparison with authentic markers. Regression analysis of the peak area was performed. The results are expressed as mg phenolic compound g^−1^ fresh weight.

### 2.7. Total Anthocyanin Content

The total anthocyanin content was measured by the pH differential method presented by Lee et al. [22]. An amount of 0.1 g of fruit samples were mixed with 20 mL buffer pH 1.0 (0.025 M potassium chloride) and pH 4 (0.4 M sodium acetate buffer), incubated for 20 min at room temperature and centrifugated at 4 °C and 7000 rpm for 15 min. Then, the absorbance of the supernatant was measured at 520 and 700 nm. To calculate the anthocyanin concentration, the following equation was used:

$$TA=\frac{A\times V}{M}$$

where *A* = *(A*_520_ nm − *A*_700_ nm) pH 1 − (*A*_520_ nm − *A*_700_ nm) pH 4.5; *V* = volume of extract (mL); and *M* = fresh mass of the sample (g). The results are expressed in *A* (absorbance) per g.

### 2.8. Total and Individual Carotenoid Compounds

The extraction of carotenoid compounds was carried out following the method described by Zacarías-García et al. [23], slightly modified. For this, 1 g of fruit samples was crushed and homogenized at 4 °C for 3 min with 12 mL of methanol/acetone/dichloromethane (25:25:50, *v*/*v*/*v*) containing 0.1% of BHT and 10 mL of water (extraction solvent). Then, the samples were sonicated in an ultrasonic water bath (P-selecta Model 516, Barcelona, Spain) for 5 min to promote the extraction of the carotenoid compounds. Subsequently, they were centrifuged at 4500× *g* for 5 min at 4 °C (Sorvall Legend XT/XF with a F13-14 × 50c carbon fiber rotor, Thermo Fischer Scientific, Cleveland, OH, USA), and the organic phase was recovered. The aqueous phase was re-extracted with 6 mL dichloromethane (HPLC grade, Sharlab, Barcelona, Spain) twice more. The organic extracts were saponified in methanolic KOH (12%, *w*/*v*) for 90 min under darkness at room temperature. After, 6 mL of 50 mM Tris-HCl pH 7.5 with 1 M NaCl and 6 mL of dichloromethane were added, stirred, and centrifuged at 4500× *g* for 5 min at 4 °C. The aqueous phase was discarded. The organic extract was filtered by Na_2_SO_4_ anhydrous, and it was dried and redissolved in 2 mL with the extraction solvent.

Total carotenoid content (TCC) was determined using a colorimetric method described by Dragovic-Uzelac et al. [24], with minor modifications. Briefly, 25 µL of the previous extract was diluted at 1 mL with extraction solvent and measured in the UV-Vis spectrophotometer (Shimadzu, Kyoto, Japan) at 450 nm. For calibration, a Zeaxanthin standard was used. The results are expressed as mg Zeaxanthin equivalents g^−1^ of fresh weight.

Individual carotenoid compounds were obtained according to Bohoyo et al. [25] by ultra-high-performance liquid chromatography (UHPLC) (Agilent 1290, Agilent Technologies, CA, USA) with a Lichrosorb RP-18 column (4.6 × 200 mm × 10 µm) thermostatically controlled at 28 °C and coupled to a DAD detector (measuring at 460 nm). The flow rate was kept constant at 0.3 mL/min. The mobile phase solvents consisted of acetonitrile/water (85:15, *v*/*v*) (phase A), and acetonitrile/methanol/ethyl acetate (60:20:20, *v*/*v*/*v*) (phase B). The gradient elution conditions were 100% phase A for 4 min, then the gradient changed to 100% phase B at 4.17 min until 9 min, and finally, the gradient changed to 100% phase A at 9.17 min until the end of the analysis (11 min). Prior to chromatographic analysis, the extract sample was filtered through a 0.22 µm filter, and 1.4 µL was injected in the chromatographic system.

### 2.9. Statistical Analysis

All analyses were performed in triplicate. Results are expressed as mean ± standard deviation. The normality of the data was tested using a Shapiro–Wilk test. When the data did not have a normal distribution, a Kruskal–Wallis with Dunn–Bonferroni test was applied. When the data presented a normal distribution, homoscedasticity was analyzed by applying Levene’s test. When data did not exhibit homoscedasticity, a Welch ANOVA test followed by a post hoc Games–Howell test was used. In cases where the data were normal and homoscedastic, an ANOVA analysis with a Tukey’s post hoc test was used. The degree of significance was set at *p* < 0.05.

On the other hand, to reduce the variables affected by the factors analyzed in this study, data were analyzed using an exploratory data model by making principal component analysis (PCA). All the analyses were performed with the XLSTAT-Pro 201,610 (Addinsoft 2009, París, France) statistical software package.

## 3. Results

### 3.1. Physicochemical Analysis

During the ripening process, the fruit undergoes continuous physicochemical changes that vary according to the species and cultivars and are associated, among others, with an increase in soluble solids and pH and a decrease in acidity [26]. Total soluble solids (TSS) and titratable acidity (TA) are important indicators of maturity of fruit. In fact, the relationship TSS/TA, defined as the maturation index (MI), is directly related to fruit taste and content of bioactive compounds [27]. Thus, the MI describes a positive correlation between the MI and consumer acceptance [28,29].

Physicochemical analysis results are shown in Table 1. TSS ranged from 15.25 to 23.00 °Brix for all species, with goji berry cultivars showing the highest values (23.00 and 21.30 °Brix for ‘Turgidus’ and ‘New Big’ cultivars, respectively) (*p* < 0.05). On the other hand, ‘Blue Velvet’ haskap showed the lowest TSS value (13.00 °Brix) (*p* < 0.05), while the aronia cultivars showed intermediate values (17.65, 17.33, and 15.25 °Brix for ‘Nero’, ‘Viking’, and ‘Galicjanka’ cultivars, respectively) (*p* < 0.05). Regarding TA, goji berries showed the lowest values (0.34–0.41 g malic acid 100 g^−1^), followed by the three aronia cultivars, with values between 0.84 and 0.96 g malic acid 100 g^−1^, while ‘Blue Velvet’ haskap is the cultivar that presents a higher acidity (3.19 g malic acid 100 g^−1^), with significant differences with respect to the goji fruits (*p* < 0.05).

The MI ranged widely among species with significant differences between them showing goji berries the highest values (68.31 and 52.50) (*p* < 0.05), while haskap berry showed the lowest one (4.07) (*p* < 0.05). Aronia fruits showed an MI ranging from 16.31 to 20.92. This is in agreement with other authors who described that more acidic cultivars generally have a lower MI at commercial maturity than less acidic cultivars regardless of changes in ripening [30].

These results suggest that goji cultivars would be better accepted by the consumer for fresh consumption. Haskap fruits would be the worst accepted by the consumer, consistent with a sweet and sour tastes wrapped in bitterness described in the literature [17]. This justifies the use of these species to obtain new products or as a source for the extraction of bioactive compounds [31,32].

### 3.2. Sugars

The content of sugars and organic acids are directly related to a fruit’s taste properties and nutritional value. The sugar contents of the different berry fruit cultivars studied are shown in Table 2. Glucose and fructose monosaccharides were the main sugars found in goji and haskap cultivars, with higher concentrations in goji (5.02 to 6.18 g 100 g^−1^ fw and 5.34 to 6.07 g 100 g^−1^ fw for fructose and glucose, respectively) with respect to aronia and haskap cultivars (*p* < 0.05). However, in aronia fruits, the main sugar was sorbitol (6.08–7.91 g 100 g^−1^ fw), which was detected in much lower amounts in haskap and goji cultivars (*p* < 0.05). These results agree with those obtained by several authors. Thus, Magro et al. [18] found that fructose and glucose were the main sugars in eight cultivars of goji berries grown in Extremadura in 2022, with concentrations ranging from 13.8 to 35.2 g 100 g^−1^ dw and 13.7 to 48.6 g 100 g^−1^ dw, respectively. However, a study of goji berry fruits grown in China [33] showed values slightly lower than those obtained in our study, ranging for fructose and glucose between 3.0 and 4.5 and between 2.0 and 3.1 g 100 g^−1^ fw, respectively. Regarding the haskap variety, fructose was the predominant sugar (3.01 g 100 g^−1^ fw), with values lower than those found by Li and Hoshino [34] (>10 g 100 g^−1^ fw) when studying the impact of the ploidy level on the accumulation of biochemical content in haskap fruits. On the other hand, the sorbitol values found in the aronia cultivars studied were similar or slightly lower than those detected by other authors in 23 samples of aronia berries grown in Bulgaria (6.55–12.99 g 100 g^−1^ fw) [35]. They suggested that the warm climate of Bulgaria, similar to the climatic conditions of our assay, could favour the accumulation of sugars such as sorbitol. These authors also found that the second most abundant sugar was fructose, followed by glucose, as in our study. Considering the sweetness intensity of each sugar, with sorbitol showing the lowest sweetening power [36], the sugar profile suggests that goji cultivars will have a higher sweetness due to higher fructose and glucose content. However, aronia cultivars will be the least sweet due to their high sorbitol content.

### 3.3. Organic Acids

In general, haskap, aronia, and goji berries are rich in organic acids, which gives them a specific sour taste, reminiscent of blueberries [37]. In our study, the organic acid contents varied according to the species. Thus, aronia and haskap fruits showed a total organic acid content (38.03–44.78 mg g^−1^ fw and 42.53 mg g^−1^ fw, respectively) much higher than that found in goji fruits (13.03–21.64 mg g^−1^ fw) (Table 2). This lower content of organic acids in goji fruits confirms the lower titratable acidity and higher pH values obtained for them (Table 1). In aronia fruits, the major organic acid was succinic (19.93–24.32 mg g^−1^ fw), followed by malic (10.55–11.84 mg g^−1^ fw), quinic (4.51–5.43 g mg^−1^ fw), and oxalic (2.13–2.22 mg g^−1^ fw) acids. However, Gerasimov et al. [38] found malic acid to be a majority in five cultivars of aronia fruits, although at lower concentrations (517.2–950.0 mg 100 g^−1^ fw) than those obtained in our study. Subsequently, they found quinic and succinic acid ranging from 396.1 to 483.7 mg 100 g^−1^ fw (approximately equal to those of our study) and from 91.9 to 240.8 mg 100 g^−1^ fw (values much lower than those of our study), respectively. On the other hand, we detected tartaric and ascorbic acids at concentrations < 1 mg g ^−1^, and citric and fumaric acids were not detected. Similarly, citric and fumaric acids were not found in haskap fruits but were found in goji berry fruits, although fumaric acid was found in an insignificant concentration (<0.05 mg g^−1^). In contrast, other authors [34] have found that citric acid is one of the predominant acids in haskap (*Lonicera caerulea* L. subsp. *edulis* (Turcz. ex Herder) Hultén) fruits. They observed a complex relationship between ploidy levels and the accumulation of organic acids, although in some cases additional factors, possibly environmental, might also play a significant role in determining the organic acid profile of the fruits [34]. In our study, the major organic acid found in haskap fruits was malic (17.38 mg g^−1^ fw) followed by tartaric (11.55 mg g^−1^ fw), quinic (10.80 mg g^−1^ fw), and succinic (1.80 mg g^−1^ fw) acids. Ascorbic and oxalic acids were found in lower amounts (0.88 and 0.12 mg g^−1^ fw, respectively). In goji berry fruits, tartaric and succinic acids were found in equivalent amounts (3.19 and 3.15 mg g^−1^ fw, respectively). Citric acid was also one of the major compounds, but with significant differences between the two goji cultivars studied (3.09 mg g^−1^ fw for ‘Turgidus’ vs. 11.8 mg g^−1^ fw for ‘New Big’). Wojdylo et al. [39] also found citric acid one of the main organic acids in goji berry fruits grown in Poland. Malic, ascorbic, and oxalic acids were found at a concentration < 3 mg g^−1^ for both cultivars.

Due to the importance of ascorbic acid or vitamin C needed by humans, we want to refer to the ascorbic acid content of the fruits studied. Ascorbic acid content in haskap berries is usually higher compared to other berries [40]. This, together with their high phenol content, makes their antioxidant effect three to five times higher than that of other commonly consumed berries, such as blackberries or strawberries [41]. In our study, goji fruits showed a higher content with respect to aronia fruits (*p* < 0.05). However, haskap fruits showed a similar content to ‘New Big’ goji cultivar (*p* > 0.05). Other authors found similar or slightly lower values than those found in our study for haskap berry cultivars grown in Switzerland (1.78–4.21 mg g^−1^ dw) [37]. Aronia fruits showed the lowest ascorbic acid content. A food product is rich in a specific nutrient when a serving provides 20% of the recommended daily intake. According to EFSA [42], the reference intake of vitamin C for adult men and women is 110 and 95 mg/day, respectively. A 100 g portion of the haskap or goji berry studied would provide more than 80 mg and 92.6 mg of ascorbic acid, respectively, so it can be stated that haskap and goji berries of the analyzed cultivars are rich in ascorbic acid.

### 3.4. Total and Individual Phenolic Content

The beneficial health effects of phenolic compounds are an argument for seeking fruits rich in these compounds. In general, berries (aronia, haskap, and goji) contain large amounts of bioactive compounds with antioxidant properties such as anthocyanins, flavonoids, and phenolic acids [43,44,45]. In addition, goji berries are considered a rich source of carotenoid compounds [18,38]. These berries are often called “superfruits” due to their potential health-promoting properties, namely antibacterial and antidiabetic effects, an ability to reduce the risk of osteoporosis, hypertension, anemia, ischemic heart disease, and gastrointestinal disorders [41]. In our study, the total phenolic content (TPC) was significantly higher in the aronia cultivars (11.75–13.94 mg GAE g^−1^ fw) compared to haskap (6.94 mg GAE g^−1^ fw) and goji (3.59–4.59 mg GAE g^−1^ fw) fruits (Table 3). In a review study, it was reported that aronia fruits can be considered a promising component of engineered foods for their antioxidant potential [46]. Ochmian et al. [47] reported slightly higher values for the same aronia cultivars (1845–2185 mg GAE 100 g^−1^), with the ‘Viking’ cultivar showing the lowest TPC as in our study. However, Zurek et al. [43] found a TPC ranging from 20.90 to 46.76 mg GAE g^−1^ fw in haskap berries (*Lonicera caerulea* var. *Kamtschatica* Sevast.) grown in Poland, much higher than the value obtained in our study (6.94 mg GAE g^−1^ fw). Regarding goji fruits, the cultivars studied in this work showed a slightly higher content than the same cultivars studied by our research team in a previous season (2.34 and 2.02 mg g^−1^ fw in ‘Turgidus’ (G3) and ‘New Big’ (G5), respectively) [18]. These results suggest the influence of harvest year on the TPC. Other authors found a slightly higher TPC (9.04 mg g^−1^ and 7.16 mg g^−1^ fw) for fresh goji fruits (*Lycium barbarum* L.) growing in Turkey and Servia, respectively [48,49]. These observed differences in the TPC may be due to the variation in species and cultivars, cultivation year, growing conditions, and different extraction and purification parameters.

With respect to the individual phenolic compounds, a total of 15 phenolic compounds were detected, although the qualitative profile depended on the species analyzed (Table 4). The largest group included hydroxycinnamic acids (chlorogenic acid—AClo, p-coumaroylquinic acid—ApC, p-coumaric—ApCou, and t-ferulic acid—t-Fer), anthocyanins (cyanidin-3-glucoside—C3G, cyanidin-3-rutinoside—C3R, and peonidin-3-rutinoside—P3R), flavan-3-ols (catechin—Cat, epicatechin—Ecat, procyanidin—PB1, and procyanidin—PB2), and flavonols (isoharmentin-3-rutinoside—I3R, kaempferol-3-rutinoside—K3R, quercetin-3-glucoside—Q3G, and quercetin-3-rutinoside—Q3R). In this work, as described by other authors, anthocyanins were the dominant group in aronia and haskap berries [9,47,50]. However, they were not detected in goji fruits because red cultivars were studied. Other black goji berry cultivars reported in the literature did show high amounts of anthocyanins [51]. The strong purple color of haskap and aronia fruits is indicative of the presence of these compounds, which have great antioxidant potential [52]. In aronia fruits, the main anthocyanin compound was C3G (1.6–1.8 mg g^−1^ fw), followed by C3R (1.2–1.3 mg g^−1^ fw), with no significant differences between cultivars (*p* > 0.05). However, in haskap fruits, C3R was the majority (4.2 mg g^−1^ fw), followed by P3R (1.3 mg g^−1^ fw), with no C3G present. Other authors did find C3G as the major anthocyanin present in haskap, although the difference may be due to the detection method used in the cyanidin identification. We can highlight that the presence of these compounds constituted more than 83% and 93% of the phenolic compounds determined in the aronia and haskap cultivars, respectively. These compounds exhibit numerous health-promoting effects including antioxidant, anti-inflammatory, cardioprotective, and anti-diabetic properties [9].

When the total anthocyanin compounds (TA) are analyzed, haskap fruits showed a significantly higher content (196.6 A g^−1^ fw) (*p* < 0.05) than aronia (111.9–123.1 A g^−1^ fw) (*p* < 0.05) and goji (not detected) fruits (Table 3). This is consistent with a higher percentage of these individual compounds (>93%) with respect to the sum of the phenolic compounds determined.

AClo, one of the main polyphenols in the human diet and associated with various health benefits [53], was found in significant amounts in aronia and goji fruits (0.165–0.371 mg g^−1^ fw), with the goji ‘New Big’ cultivar showing the highest content (*p* < 0.05). However, it was not detected in haskap fruits, although other authors did find it in the range of 0.86 to 2.67 mg g^−1^ [54] and 0.22 to 0.46 mg g^−1^ [55]. These differences in phenolic acid content have been found to vary in accordance with the cultivar growing conditions and time of harvest [46]. Other compounds also of high health importance for their antitumor properties [56], such as Q3G and Q3R, were found in higher amounts in haskap and aronia compared to goji fruits (Table 3). In particular, haskap fruits showed the significantly higher content of Q3R (0.160 mg g^−1^ fw) (*p* < 0.05).

### 3.5. Total and Individual Carotenoid Compounds

Carotenoids are important color pigments that contribute to human health [57]. In general, haskap and aronia fruits are purplish blue in color with very low amounts of carotenoids [58], usually less than 0.05 mg g^−1^ [7,46]. However, goji fruits have a significant content of carotenoid compounds due to their orange–red color [18,39]. In this work, the total carotenoid content (TCC) as well as individual carotenoid compounds were not detected in aronia and haskap fruits (Table 3). Nevertheless, goji fruits showed a TCC ranging from 0.27 to 0.37 mg Zea g^−1^ fw. These values confirm those found by the research team for goji fruits growing on the same site but in different production seasons [18]. Regarding the carotenoid profile, capsanthin (Cap), zeaxanthin (Zea), β-cryptoxanthin (β-Crp), α-carotene (α-Car), and β-carotene (β-Car) were identified, with Zea being the carotenoid with significantly higher concentrations (39.3 and 85.7 μg g^−1^ fw for ‘Turgidus’ and ‘New Big’, respectively). Cap and β-Car followed but in much lower concentrations (1.5 to 2.5 μg g^−1^ fw for Cap and 1.1 to 1.8 μg g^−1^ fw for β-Car) than Zea (Table 4). Although the literature shows large variation in carotenoid contents between cultivars, Zea was the majority in goji fruits [14,18].

### 3.6. Antioxidant Activity

The antioxidant capacity values of the fruits analyzed by the DPPH method are shown in Table 3. Aronia fruits showed the highest antioxidant capacity (12.0–15.9 g Trolox kg^−1^ fw) compared to haskap (6.70 g Trolox kg^−1^ fw) and goji (5.33–5.69 g Trolox kg^−1^ fw) fruits. This is consistent with a higher content of the TPC in aronia fruits. Goji fruits of the ‘Turgidus’ cultivar showed a significantly lower value (*p* < 0.05) according to a significantly lower TPC (*p* < 0.05). In the same sense, other authors reported that the antioxidant activity of berries depends mainly on their chemical composition, in particular the content and varied structure of polyphenolic compounds that influence their antioxidant potential [47,59].

A correlation study between ascorbic acid, TPC, AT, TCC, individual phenolic and carotenoid compound, and AAT values based on the Pearson correlation coefficients was investigated (Table 4). High correlations were found mainly with the TPC (0.96) and other individual phenolic compounds such as C3G (0.91), K3R (0.88), ApC (0.68), PB2 (0.68), I3R (0.66), and Q3G (0.66) (*p* < 0.05). However, ascorbic acid and total and individual carotenoid compounds showed a significant negative correlation with the AAT. These results confirm that phenolic compounds are the main contributors to the antioxidant activity of berries, as already described by other authors [50,60,61].

### 3.7. PCA Analysis

In order to understand the interrelationships between the studied variables and the results of the different samples, a principal component analysis was carried out, which helps to clarify the results. The first two components accounted for 87.78% of the total variance. The proportion of the variance explained by the first component, PC1, was 60.03% of the total variance, and PC1 is constituted by ApCou, different variables related to carotenoids (TCC, α-Car, and Crp), pH, and glucose and fructose, with factor loadings positive and higher than 0.9; in the negative part of the axis, the variables are the phenolic compounds PB2 and Q3R and malic acid (Table 5). PC2, accounted for 27.76% of the total variance, and the variables with higher loadings were tartaric acid; the phenolic compounds P3R, PB1, and t-Fer were in the positive part of the axis and AClo and succinic acids in the negative one (Table 5).

**Figure 1:**
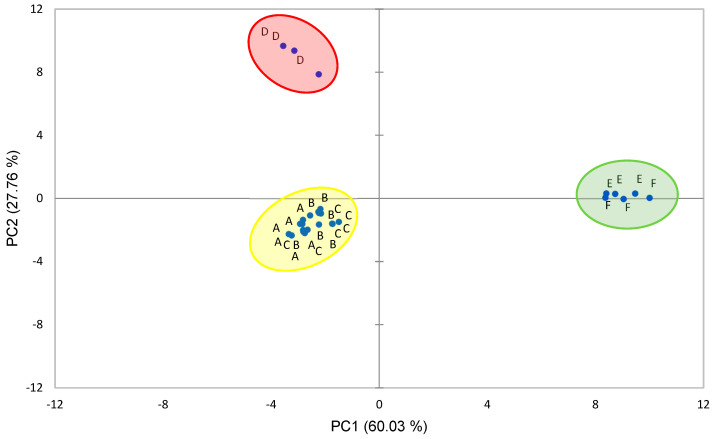
Principal component analysis (PCA) plotting components 1 and 2 for the different berry cultivars (A: ‘Nero’, B: ‘Viking’, C: ‘Galicjanka’, D: ‘Blue Velvet’, E: ‘Turgidus’, and F: ‘New Big’). (PC1: 60.03% of total variance; PC2: 27.76% of total variance).

When the samples are represented on the plane defined by the first two principal components (Figure 1), different positions can be observed depending on the berry species and cultivars. The clear separation of the three berry species in the study on the PCA plane is due to their chemical composition. On the positive part of the PC1 axis are the samples of the two goji berry cultivars due to the high concentrations of carotenoid compounds and sugars as well as higher pH and p-coumaroylquinic acid values. The aronia and haskap berry cultivars occupy a position in the negative zone of this axis, with PC1 values between −1.5 and −3.5. These two species also occupy different positions in the plane. PC2 values were positive for haskap berries, related to the tartaric acid, P3R, PB1, and t-Fer, and were negative in the three aronia cultivars, being influenced mainly by AClo and succinic acid.

## 4. Conclusions

In this study, different cultivars of aronia, haskap, and goji species were analyzed, highlighting important aspects related to their physicochemical and bioactive quality. The results suggested that goji cultivars would be better accepted by the consumer for fresh consumption due to a lower content of organic acids and a higher presence of sugars, such as fructose and glucose, with higher sweetening power. However, haskap and aronia fruits would be less accepted, consistent with a bitter taste described in the literature. This justifies the use of these species mainly to obtain new products or as a source for bioactive compound extraction. On the other hand, all cultivars showed a high presence of bioactive compounds, being anthocyanins, indicative of the purple color of the fruits and with high antioxidant power, the majority of which was in haskap and aronia. However, goji cultivars showed high contents of carotenoid compounds, indicative of the red–orange color of the fruits.

These findings confirm the bioactive potential of these crops adapted to a mountainous region of central-western Spain, providing an opportunity for diversification in these harsh agricultural areas and greater resilience to social and climatic changes.

## Figures and Tables

**Table 1:** Physicochemical parameters of berry fruit cultivars expressed as mean ± SD. TSS = Total soluble solids; TA = titratable acidity.

	Cultivar	Moisture (%)	TSS (°Brix)	pH	TA (%)	MI
Aronia	‘Nero’	77.71 ± 1.19 ^bc^	17.65 ± 0.50 ^c^	3.83 ± 0.04 ^abc^	0.85 ± 0.07 ^abc^	20.92 ± 1.43 ^b^
	‘Viking’	78.55 ± 0.36 ^abc^	17.33 ± 0.37 ^c^	3.80 ± 0.02 ^abc^	0.84 ± 0.04 ^abc^	20.58 ± 0.93 ^b^
	‘Galicjanka’	80.65 ± 0.81 ^ab^	15.25 ± 0.30 ^d^	3.77 ± 0.01 ^bc^	0.96 ± 0.20 ^ab^	16.31 ± 2.69 ^b^
Haskap	‘Blue Velvet’	86.45 ± 0.19 ^a^	13.00 ± 0.35 ^e^	3.23 ± 0.01 ^c^	3.19 ± 0.07 ^a^	4.07 ± 0.18 ^c^
Goji	‘Turgidus’	75.71 ± 1.06 ^c^	23.00 ± 0.96 ^a^	5.56 ± 0.03 ^a^	0.34 ± 0.02 ^c^	68.31 ± 2.83 ^a^
	‘New Big’	77.98 ± 2.62 ^abc^	21.30 ± 0.53 ^b^	5.33 ± 0.01 ^ab^	0.41 ± 0.02 ^bc^	52.50 ± 1.17 ^a^

Different superscript letters in the same row indicate significant differences among berry fruit cultivars. Multiple comparisons were analyzed following a Kruskal–Wallis test (*p* < 0.05) for moisture, pH, TA, and RI and a Tukey test (*p* < 0.05) for TSS.

**Table 2:** Sugar and organic acid composition of berry fruit cultivars expressed as mean ± SD.

	Aronia			Haskap	Goji	
Cultivar	‘Nero’	‘Viking’	‘Galicjanka’	‘Blue Velvet’	‘Turgidus’	‘New Big’
Sugars (g 100 g^−1^ fw)						
Fructose	2.62 ± 0.71 ^b^	2.43 ± 0.57 ^b^	2.91 ± 0.36 ^b^	3.01 ± 0.46 ^b^	6.18 ± 0.18 ^a^	5.02 ± 0.53 ^a^
Glucose	2.63 ± 0.38 ^b^	1.96 ± 0.60 ^b^	2.40 ± 0.18 ^b^	2.44 ± 0.38 ^b^	6.07 ± 0.18 ^a^	5.34 ± 0.57 ^a^
Sorbitol	6.37 ± 0.15 ^b^	7.91 ± 0.79 ^a^	6.08 ± 0.52 ^b^	0.48 ± 0.12 ^c^	n.d.	0.42 ± 0.06 ^c^
Total	11.62 ± 0.96 ^a^	12.30 ± 0.99 ^a^	11.39 ± 1.02 ^a^	5.93 ± 0.79 ^b^	12.24 ± 0.37 ^a^	10.79 ± 1.13 ^a^
Organic Acids (mg g^—1^ fw)						
Oxalic	2.15 ± 0.25 ^a^	2.13 ± 0.34 ^a^	2.22 ± 0.51 ^a^	0.12 ± 0.01 ^a^	0.09 ± 0.01 ^a^	0.18 ± 0.01 ^a^
Citric	n.d.	n.d.	n.d.	n.d.	3.09 ± 0.27 ^b^	11.8 ± 1.17 ^a^
Tartaric	0.70 ± 0.05 ^ab^	0.60 ± 0.02 ^b^	0.60 ± 0.04 ^b^	11.55 ± 0.34 ^a^	3.19 ± 0.24 ^ab^	3.82 ± 0.42 ^ab^
Malic	11.84 ± 0.36 ^ab^	10.55 ± 0.31 ^ab^	10.91 ± 0.83 ^ab^	17.38 ± 0.92 ^a^	2.22 ± 0.12 ^b^	1.47 ± 0.07 ^b^
Quinic	5.43 ± 0.23 ^b^	4.51 ± 0.28 ^b^	4.58 ± 0.33 ^b^	10.80 ± 0.81 ^a^	n.d.	n.d.
Ascorbic	0.34 ± 0.02 ^cd^	0.31 ± 0.02 ^d^	0.29 ± 0.04 ^d^	0.88 ± 0.05 ^bc^	1.29 ± 0.09 ^a^	1.01 ± 0.10 ^ab^
Succinic	24.32 ± 0.65 ^a^	19.93 ± 0.47 ^a^	21.40 ± 1.07 ^a^	1.80 ± 0.12 ^b^	3.15 ± 0.18 ^b^	3.37 ± 0.31 ^b^
Fumaric	n.d.	n.d.	n.d.	n.d.	0.011 ± 0.002 ^a^	0.003 ± 0.002 ^b^
Total	44.78 ± 1.33 ^a^	38.03 ± 0.85 ^ab^	40.00 ± 2.80 ^ab^	42.53 ± 2.18 ^ab^	13.03 ± 0.88 ^b^	21.64 ± 2.08 ^ab^

n.d.: Not detected. Different superscript letters in the same row indicate significant differences among berry fruit cultivars. Multiple comparisons were analyzed following a Tukey test (*p* < 0.05) for sugars; a Kruskal–Wallis test (*p* < 0.05) for oxalic, tartaric, malic, quinic, ascorbic, succinic, and total acids; and a Student’s t-test (*p* < 0.05) for citric and fumaric acids.

**Table 3:** Total and individual phenolic contents, antioxidant activity, total anthocyanins, and total and individual carotenoid contents of goji berries expressed as mean ± SD.

Species	Aronia			Haskap	Goji	
Cultivars	‘Nero’	‘Viking’	‘Galicjanka’	‘Blue Velvet’	‘Turgidus’	‘New Big’
Total Phenolic Compounds (TPCs) (mg GAE g^−1^ fw)	13.94 ± 1.04 ^a^	11.75 ± 2.56 ^ab^	13.82 ± 2.09 ^ab^	6.94 ± 0.20 ^ab^	3.59 ± 0.82 ^b^	4.59 ± 1.61 ^ab^
Individual Phenolic Compounds (mg g^−1^ fw)						
Chlorogenic acid (AClo)	0.29 ± 0.02 ^b^	0.26 ± 0.02 ^b^	0.198 ± 0.003 ^c^	n.d.	0.165 ± 0.007 ^c^	0.37 ± 0.02 ^a^
p-coumaroylquinic acid (ApC)	0.05 ± 0.02 ^a^	0.014 ± 0.003 ^ab^	0.012 ± 0.001 ^ab^	n.d.	0.003 ± 0.001 ^b^	0.007 ± 0.001 ^ab^
p-coumaric acid (ApCou)	n.d.	n.d.	n.d.	n.d.	0.0021 ± 0.0003 ^a^	0.0021 ± 0.0002 ^a^
t-ferulic acid (t-Fer)	0.0032 ± 0.0007 ^a^	0.0030 ± 0.0006 ^a^	0.0043 ± 0.0005 ^a^	0.010 ± 0.002 ^a^	0.0030 ± 0.0001 ^a^	0.0056 ± 0.0004 ^a^
Cyanidin-3-glucoside (C3G)	1.8 ± 0.1 ^a^	1.8 ± 0.2 ^a^	1.6 ± 0.2 ^a^	0.004 ± 0.001 ^b^	n.d.	n.d.
Cyanidin-3-rutinoside (C3R)	1.2 ± 0.1 ^b^	1.3 ± 0.2 ^b^	1.3 ± 0.1 ^b^	4.2 ± 0.7 ^a^	n.d.	n.d.
Peonidin-3-rutinoside (P3R)	n.d.	n.d.	n.d.	1.3 ± 0.1	n.d.	n.d.
Catechin (Cat)	n.d.	n.d.	n.d.	0.030 ± 0.009 ^a^	0.037 ± 0.005 ^a^	0.066 ± 0.002 ^a^
Epicatechin (Ecat)	0.044 ± 0.002 ^ab^	0.037 ± 0.008 ^ab^	0.029 ± 0.003 ^b^	0.07 ± 0.01 ^a^	n.d.	n.d.
Procyanidin B1 (PB1)	n.d.	n.d.	n.d.	0.021 ± 0.005	n.d.	n.d.
Procyanidin B2 (PB2)	0.019 ± 0.002 ^a^	0.015 ± 0.004 ^a^	0.014 ± 0.003 ^a^	0.020 ± 0.003 ^a^	n.d.	n.d.
Isoharmentin-3-rutenoside (I3R)	0.006 ± 0.001 ^a^	0.004 ± 0.001 ^a^	0.004 ± 0.001 ^a^	0.004 ± 0.001 ^a^	n.d.	0.004 ± 0.001 ^a^
Kaempferol-3-rutinoside (K3R)	0.008 ± 0.001 ^a^	0.006 ± 0.001 ^a^	0.007 ± 0.001 ^a^	0.003 ± 0.001 ^a^	n.d.	n.d.
Quercetin-3-glucoside (Q3G)	0.062 ± 0.001 ^a^	0.051 ± 0.005 ^a^	0.054 ± 0.004 ^a^	0.069 ± 0.014 ^a^	n.d.	n.d.
Quercetin-3-rutinoside (Q3R)	0.088 ± 0.005 ^ab^	0.07 ± 0.01 ^ab^	0.087 ± 0.006 ^ab^	0.16 ± 0.04 ^a^	0.028 ± 0.003 ^ab^	0.017 ± 0.002 ^b^
Total anthocyanins (TA) (A g^−1^ fw)	112.0 ± 12.2 ^b^	120.6 ± 26.1 ^b^	123.1 ± 30.5 ^b^	196.6 ± 18.0 ^a^	n.d.	n.d.
Total carotenoid contents (TCCs) (mg Zea g^−1^ fw)	n.d.	n.d.	n.d.	n.d.	0.27 ± 0.03 ^a^	0.37 ± 0.05 ^a^
Individual carotenoids (μg g^−1^ fw)						
Capsanthin (Cap)	n.d.	n.d.	n.d.	n.d.	1.5 ± 0.1 ^a^	2.5 ± 0.7 ^a^
Zeaxanthin (Zea)	n.d.	n.d.	n.d.	n.d.	39.3 ± 14.8 ^a^	85.7 ± 31.9 ^a^
Cryptoxanthin (Crp)	n.d.	n.d.	n.d.	n.d.	0.8 ± 0.2 ^a^	1.1 ± 0.3 ^a^
α-carotene (α-Car)	n.d.	n.d.	n.d.	n.d.	0.4 ± 0.1 ^a^	0.4 ± 0.1 ^a^
β-carotene (β-Car)	n.d.	n.d.	n.d.	n.d.	1.1 ± 0.4 ^a^	1.8 ± 0.5 ^a^
Antioxidant activity (AAT) (g Trolox kg^−1^ fw)	15.9 ± 1.2 ^a^	12.0 ± 2.4 ^ab^	13.33 ± 1.03 ^ab^	6.7 ± 0.5 ^ab^	5.3 ± 0.2 ^b^	5.7 ± 0.2 ^ab^

n.d.: Not detected; different superscript letters in the same row indicate significant differences among berry fruit cultivars. Multiple comparisons were analyzed following a Tukey test (*p* < 0.05) for AT and AClo; a Kruskal–Wallis test (*p* < 0.05) for the TPC, ApC, t-Fer, C3G, C3R, Cat, Ecat, PB2, I3R, K3R, Q3G, Q3R, and AAT; and a Student’s *t*-test (*p* < 0.05) for ApCou and total and individual carotenoids.

**Table 4:** Correlation matrix (Pearson) of total and individual phenolic, anthocyanins, and carotenoid compounds and antioxidant activity.

	AAT	AA	TPC	TA	TCC	Cap	Zea	Crp	α-Car	β-Car	AClo	ApC	ApCou	t-Fer	C3G	C3R	P3R	Cat	Ecat	PB1	PB2	I3R	K3R	Q3G	Q3R
AAT	1 *																								
AA	−0.86 *	1																							
TPC	0.96 *	0.89 *	1 *																						
TA	0.47 *	−0.57 *	0.58 *	1 *																					
TCC	−0.71 *	0.81 *	−0.77 *	−0.86 *	1 *																				
Cap	−0.67 *	0.79 *	−0.72 *	−0.82 *	0.98 *	1 *																			
Zea	−0.62 *	0.69 *	−0.65 *	−0.76 *	0.92 *	0.96 *	1 *																		
β-Crp	−0.69 *	0.80 *	−0.74 *	−0.84 *	0.95 *	0.94 *	0.95 *	1 *																	
α-Car	−0.70 *	0.84 *	−0.76 *	−0.85 *	0.93 *	0.89 *	0.89 *	0.98 *	1 *																
β-Car	−0.67 *	0.75 *	−0.71 *	−0.81 *	0.94 *	0.94 *	0.97 *	0.99 *	0.95 *	1 *															
AClo	0.29	−0.25	0.18	−0.49 *	0.29	0.33	0.36	0.27	0.19	0.32	1 *														
ApC	0.68 *	−0.46 *	0.52 *	0.13	−0.32	−0.30	−0.28	−0.32	−0.33	−0.30	0.47 *	1 *													
ApCou	−0.72 *	0.85 *	−0.79 *	−0.87 *	0.97 *	0.92 *	0.86 *	0.96 *	0.98 *	0.92 *	0.22	−0.34	1 *												
t-Fer	−0.43 *	0.336	−0.32	0.33	0.02	0.05	0.09	0.01	−0.03	0.05	−0.62 *	−0.39 *	−0.03	1 *											
C3G	0.91 *	−0.94 *	0.89 *	0.41 *	−0.73 *	−0.69 *	−0.65 *	−0.72 *	−0.73 *	−0.69 *	0.35	0.53 *	−0.74 *	−0.55 *	1 *										
C3R	0.01	−0.14	0.12	0.80 *	−0.58 *	−0.55 *	−0.52 *	−0.57 *	−0.58 *	−0.55 *	−0.74 *	−0.12	−0.59 *	0.76 *	−0.06	1 *									
P3R	−0.38	0.30	−0.29	0.53 *	−0.18	−0.18	−0.16	−0.18	−0.18	−0.17	−0.79 *	−0.31	−0.19	0.89 *	−0.49 *	0.89 *	1 *								
Cat	−0.83 *	0.86 *	−0.84 *	−0.61 *	0.89 *	0.89 *	0.87 *	0.88 *	0.83 *	0.88 *	0.02	−0.43 *	0.85 *	0.43 *	−0.90 *	−0.18	0.23	1 *							
Ecat	0.37	−0.42 *	0.42 *	0.87 *	−0.78 *	−0.74 *	−0.69 *	−0.77 *	−0.77 *	−0.74 *	−0.51 *	0.23	−0.79 *	0.49 *	0.29	0.91 *	0.67 *	−0.46 *	1 *						
PB1	−0.38	0.30	−0.28	0.51 *	−0.18	−0.17	−0.16	−0.18	−0.18	−0.17	−0.78 *	−0.31	−0.19	0.91 *	−0.48 *	0.89 *	0.99 *	0.24	0.68 *	1 *					
PB2	0.68 *	−0.70 *	0.71 *	0.82 *	−0.90 *	−0.86 *	−0.80 *	−0.88 *	−0.89 *	−0.86 *	−0.26	0.43 *	−0.91 *	0.16	0.63 *	0.68 *	0.32	−0.72 *	0.91 *	0.33	1 *				
I3R	0.66 *	−0.69 *	0.61 *	0.48 *	−0.56 *	−0.47 *	−0.39 *	−0.57 *	−0.66 *	−0.49 *	0.33	0.67 *	−0.65 *	0.04	0.59 *	0.31	0.01	−0.44 *	0.58 *	0.04	0.71 *	1 *			
K3R	0.88 *	−0.92 *	0.89 *	0.56 *	−0.86 *	−0.82 *	−0.76 *	−0.84 *	−0.85 *	−0.81 *	0.14	0.53 *	−0.87 *	−0.35	0.92 *	0.21	−0.22	−0.91 *	0.53 *	−0.22	0.81 *	0.70 *	1 *		
Q3G	0.66 *	−0.74 *	0.71 *	0.88 *	−0.94 *	−0.89 *	−0.84 *	−0.92 *	−0.93 *	−0.89 *	−0.30	0.38	−0.95 *	0.21	0.63 *	0.72 *	0.35	−0.75 *	0.90 *	0.36	0.97 *	0.71 *	0.80 *	1 *	
Q3R	0.27	−0.34	0.35	0.84 *	−0.73 *	−0.71 *	−0.66 *	−0.71 *	−0.71 *	−0.67 *	−0.66 *	0.07	−0.73 *	0.63 *	0.18	0.95 *	0.74 *	−0.39 *	0.95 *	0.76 *	0.82 *	0.46 *	0.42 *	0.86 *	1 *

* Correlation is significant at *p* < 0.05. AAT: antioxidant activity; AA: ascorbic acid; TPC: total phenolic content; TA: total anthocyanins; TCC: total carotenoid content; AClo: chlorogenic acid; ApC: p-coumaroylquinic acid; ApCou: p-coumaric acid; t-Fer: t-ferulic acid; C3G: cyanidin-4-rutinoside; C3R: cyanidin-3-rutinoside; P3R: peonidin-3-rutinoside; Cat: catechin; Ecat: epicatechin; PB1: procyanidin B1; PB2: procyanidin B2; I3R: Isoharmentin-3-rutinenoside; K3R: kaempferol-3-rutinoside; Q3G: quercetin-3-glucoside; Q3R: quercetin-3-rutinoside; Cap: Capsanthin; Zea: zeaxanthin; β-Crp: β-cryptoxanthin; α-Car: α-carotene; β-Car: β-carotene.

**Table 5:** Factor loadings PC1 and PC2.

	Component			
	1		2	
Positive	ApCou	0.988	Tartaric Acid	0.977
	TCC	0.977	P3R	0.968
	α-Car	0.975	PB1	0.963
	Crp	0.969	t-Fer	0.914
	pH	0.967		
	Glucose	0.954		
	Cap	0.938		
	β-Car	0.938		
	Fructose	0.921		
Negative	Q3R	0.975	AClo	0.799
	PB2	0.947	Succinic Acid	0.703
	Malic Acid	0.915		

## Data Availability

The original contributions presented in the study are included in the article; further inquiries can be directed to the corresponding author.
